# Zinc

**DOI:** 10.1016/j.advnut.2025.100408

**Published:** 2025-03-12

**Authors:** Liping Huang, Carmen P Wong, Emily Ho

**Affiliations:** 1 USDA/Agricultural Research Service/Western Human Nutrition Research Center, Department of Nutrition, University of California, Davis, CA, United States; 2 Linus Pauling Institute and School of Nutrition and Public Health, College of Health, Corvallis, OR, United States

Zinc was recognized as an essential trace metal for humans in the early 1960s. Zinc metal existing as Zn^2+^ is a strong electron acceptor in biological systems without risk of causing oxidant damage to cells. Zn^2+^ functions in the structure of proteins and is a catalytic component of >300 different enzymes, encompassing almost every aspect of biology, including growth, immune defense, cognitive function, and bone health. Because zinc plays such a fundamental role in the survival of organisms, its concentration in the body must be adequate and well controlled.

Zinc homeostasis is maintained by 2 families of zinc transporters, SLC30A [zinc transporter (ZNTs)] and SLC39A [Zrt-, Irt-like proteins (ZIPs)], as well as metallothioneins. In humans, the SLC30A family includes 10 members that transport zinc ions across cell membranes into the extracellular space or move zinc ions across organelle membranes from the cytosol into the organelle. The SLC39A family has 14 members that function in an opposite direction of the SLC30A proteins. Metallothioneins are small cysteine-rich metalloproteins that tightly bind to both essential minerals like zinc and to heavy metal ions, protecting cells from metal toxicity. These proteins work together in concert to balance dietary zinc uptake and endogenous zinc excretion, maintaining cellular zinc concentrations within a narrow physiological range.

## Deficiencies

Zinc deficiency can result from low dietary intake of zinc, inadequate zinc absorption, increased zinc excretion, or an increased need for zinc (for example, children and pregnant women). Zinc deficiency is associated with stunted growth, poor appetite, dermatitis, alopecia, hypogonadism, and impaired immune function that can lead to frequent diarrhea and/or upper respiratory tract infection. Severe zinc deficiency caused by low dietary zinc intake is uncommon in the developed world; however, it is seen in patients with inherited (genetic defects in zinc transporter genes) or acquired conditions. Several rare genetic disorders have been described in humans, including acrodermatitis enteropathica (AE), transient neonatal zinc deficiency, and Ziegler–Huang syndrome (bone marrow failure syndrome 8). Patients with AE experience severe zinc deficiency from birth because of mutations in *SLC39A4* (*ZIP4*), which encodes a major intestinal zinc uptake protein. Infants who are breastfed by lactating mothers carrying mutations in *SLC30A2* (*ZNT2*) suffer transient neonatal zinc deficiency due to inadequate zinc supply by breast milk. Additionally, children carrying mutations in *SLC30A7* (*ZNT7*) undergo low serum zinc concentration accompanied with stunted growth, delayed bone age, hypogonadism, and bone marrow failure. Zinc deficiency can also result from certain malabsorption syndromes and from conditions that increase the excretion of the mineral, such as prolonged diarrhea. Moreover, zinc deficiency has been seen in those undergoing total parenteral nutrition without adequate zinc and in those with alcohol use disorder.

A milder form of zinc deficiency may be relatively common worldwide; however, the lack of a sensitive and specific biomarker of zinc status hinders the scientific study of marginal zinc deficiency in human health. Individuals at risk of zinc deficiency are listed in Table 1.

**TABLE 1:** Individuals at risk of zinc deficiency.

Individuals at risk of zinc deficiency
Malnourished individuals
Individuals with severe or persistent diarrhea
Premature or low-birth-weight infants
Children and adolescents
Pregnant and lactating women
Individuals with malabsorption syndromes
Individuals with inflammatory bowel disease (Crohn’s disease, ulcerative colitis)
Patients with chronic kidney disease, especially those on hemodialysis
Individuals with sickle cell disease
Patients who have undergone bariatric surgery
People with alcohol use disorder
Vegetarians (especially vegans)
Adults ≥ 65 y of age

## Dietary Recommendations

The dietary requirements of zinc are based on the amount of dietary zinc needed to match endogenous excretion of the mineral and are calculated through the use of a factorial modeling approach. The daily intake requirement, the estimated average requirement (EAR), was used to establish the recommended daily allowance (RDA) and was recently reviewed in the USDA Scientific Report of the 2020 Dietary Guidelines Advisory Committee. The EAR and RDA recommendations are presented in Table 2 by age and gender group [1].

**TABLE 2:** DRIs for zinc (mg/d).

	EAR (mg/d)		RDA (mg/d)		AI	UL
	Males	Females	Males	Females		
Age						
0–6 mo	—	—	—	—	2	4
7–12 mo	2.5	2.5	3	3	—	5
1–3 y	2.5	2.5	3	3	—	7
4–8 y	4	4	5	5	—	12
9–13 y	7	7	8	8	—	23
14–18 y	8.5	7.3	11	9	—	34
≥19 y	9.4	6.8	11	8	—	40
Pregnant, ≤18 y	—	10	—	12	—	34
Pregnant, 19–50 y	—	9.5	—	11	—	40
Lactating, ≤18 y	—	10.9	—	13	—	34
Lactating, 19–50 y	—	10.4	—	12	—	40

Abbreviations: AI, adequate intake; DRI, Dietary reference intake; EAR, estimated average requirement; RDI, recommended daily allowance; UL, tolerable upper intake level.

## Food Sources

Zinc is present in a wide variety of foods, with rich sources being shellfish and red meat. Zinc bioavailability from animal sources is relatively high because of the absence of compounds that inhibit its absorption and the presence of certain amino acids that enhance absorption. Beans, nuts, whole-grain products, and fortified foods (like fortified cereals) are also good sources of zinc, although zinc bioavailability from plant sources is lower because of the presence of phytic acid, which binds zinc and inhibits its absorption.

## Clinical Uses

### Diagnosis of zinc deficiency

Zinc deficiency is diagnosed by clinical manifestations, such as dermatitis, alopecia, poor appetite, frequent diarrhea and/or upper respiratory infection, stunted growth in children, and hypogonadism. Low serum zinc concentration is an indicator of zinc deficiency, albeit not a sensitive one.

### Diarrhea (children)

Zinc supplementation helps reduce the severity and duration of diarrhea in poorly nourished children who are usually zinc deficient.

### Acrodermatitis enteropathica

Zinc supplementation is the only effective treatment of this fatal disease. Patients with AE are treated with either oral or intravenous zinc, depending on the severity of zinc deficiency, and require lifelong therapeutic zinc supplementation. The major side effect of long-term, high-dose oral zinc supplementation is zinc-induced copper deficiency and copper deficiency-induced anemia caused by zinc interfering with dietary copper absorption.

### Transient neonatal zinc deficiency

Zinc supplementation to affected infants is the only effective treatment or infants are switched to formula fed.

### Ziegler–Huang syndrome

Treatment includes a combination of zinc supplementation with growth hormone and steroid hormone treatments through childhood to adolescence and sex hormone treatment during puberty. If not treated, the patient can die because of bone marrow failure.

### Wilson’s disease

Wilson’s disease is a genetic disorder of copper metabolism. Patients with Wilson’s disease have a defect in endogenous copper excretion. Thus, copper accumulates in tissues, such as the liver and brain, causing neurological or psychiatric symptoms and liver disease. Therapeutic zinc supplementation has been used for the treatment of Wilson’s disease because zinc can compete with copper for absorption in the gut.

### Patients with kidney failure

Regular hemodialysis results in endogenous zinc loss. Zinc supplements, along with other minerals like copper and iron, are commonly prescribed to hemodialysis patients.

### Other conditions

Zinc supplementation is used in patients with severe burns to promote wound healing, in patients with sickle cell disease to reduce pain crises and complications from infections, and in patients with age-related macular degeneration to slow disease progression.

## Toxicity

Zinc toxicity is rare because of the homeostatic regulation of body zinc concentrations at sites of dietary zinc absorption (small intestine) and endogenous zinc excretion (pancreatic exocrine acini, intestinal tract, and kidneys). However, long-term zinc supplementation over the tolerable upper intake level (40 mg/d for adults) can interfere with copper absorption and cause copper deficiency.

## Recent Research

Zinc has been implicated in many aspects of human biology and is important in maintaining human health [2]. Here, we highlight the recent progress of zinc research, with focus on zinc status and zinc supplementation in infectious diseases, diabetes, cancer, and neurological diseases.

### Zinc, immunity, and infectious diseases

Zinc is essential for normal development and function of the immune system because zinc is a cofactor for many proteins involved in immune regulation. Zinc deficiency negatively impacts multiple facets of immunity, resulting in impaired or dysregulated immune response and increased susceptibility to infectious diseases [3]. In humans, zinc supplementation lowers the incidence, duration, symptoms, and recovery times in infectious diseases encompassing viral (common cold and hepatitis C), bacterial (tuberculosis and pneumonia), and parasitic (malaria and Leishmania) pathogens. During the COVID-19 pandemic, there was increased interest in the potential beneficial effects of zinc supplementation in COVID-19-infected individuals. Zinc supplementation resulted in reduced disease severity, symptoms, and mortality in some, but not all studies [4,5]. The heterogeneous study outcome is likely attributed to variations in the health status of participants, dosage and formula of zinc, duration of supplementation, as well as baseline zinc status of individuals.

### Zinc and diabetes

The insulin-producing pancreatic β-cell, which contains some of the highest concentrations of zinc in the body, functions in insulin synthesis, maturation, secretion, and signaling. Decreased pancreatic zinc concentrations have been found in patients with diabetes and in mouse models of type 2 diabetes (T2D). ZNT8 is responsible for transporting zinc into the insulin secretory granule, and genome-wide association studies have identified variants in *ZNT8* with an increased risk of T2D. Low zinc status is associated with impaired insulin secretion, decreased insulin sensitivity, and increased inflammatory biomarkers in T2D. The benefits of zinc supplementation in improving glycemic control and cardiometabolic health in patients with T2D can be important in disease prevention and management. Meta-analyses of randomized controlled trials showed zinc supplementation improved glycemic control, insulin sensitivity, and reduced high-sensitivity C-reactive protein inflammatory marker in patients with diabetes and patients at high risk of developing diabetes [6]. Zinc supplementation also reduced cardiometabolic risk factors, including improving triglycerides, total cholesterol, and LDL cholesterol.

### Zinc and cancer

In addition to the function of zinc in antioxidant and DNA damage defense, zinc is critical in mediating immune response, inflammation, cell signaling, apoptosis, and cell invasion. Collectively, zinc plays a crucial role in anti-cancer defense mechanisms [7]. Decreases in serum zinc concentrations are associated with a wide variety of cancers, including head and neck, lung, breast, liver, stomach, ovarian, colon, and prostate cancer. Zinc supplementation has shown promise in reducing the risk of developing liver and breast cancer and may have potential as an adjuvant therapy for patients with cancer. More work needs to be done to determine the benefits of increasing zinc intake for cancer protection.

### Zinc and neurological diseases

Zinc plays an important role in the development and function of the central nervous system, including neurogenesis, neuronal synaptic activity and plasticity, redox homeostasis, and immune modulation [8]. Rare genetic defects in zinc transporters, such as SLC30A9 and SLC39A5, cause neuropathological changes in affected individuals albeit without showing serum zinc deficiency. On the other hand, zinc deficiency is associated with a variety of neurological disorders, including Alzheimer’s disease, Parkinson’s disease, traumatic brain injury, epilepsy, and depression. Meta-analyses of randomized controlled trials showed zinc supplementation, as a monotherapy or in combination with antidepressant medications, decreased risk of clinical depression [9,10].

This article is an updated version of a prior publication about this nutrient [11].

## Author contributions

All authors read and approved the final manuscript.

## Funding

The authors reported no funding received for this study.

## Conflict of interest

EH serves on the Scientific Advisory Boards for Haleon, Vytology, and Amway. EH has served as a paid scientific expert for testimony with Bayer. All other authors report no conflicts of interest.
